# Scandium, yttrium, and lanthanide occurrence in *Cantharellus cibarius* and *C. minor* mushrooms

**DOI:** 10.1007/s11356-023-25210-6

**Published:** 2023-01-12

**Authors:** Małgorzata Mędyk, Jerzy Falandysz, Innocent Chidi Nnorom

**Affiliations:** 1 Environmental Chemistry & Ecotoxicology, University of Gdańsk, 63 Wita Stwosza Str., 80-308 Gdańsk, PL Poland; 2 Department of Toxicology, Faculty of Pharmacy, Medical University of Lodz, 1 Muszyńskiego Street, 90-151 Łódź, Poland; 3 Analytical/Environmental Unit, Department of Pure and Industrial Chemistry, Abia State University, Uturu, Nigeria

**Keywords:** Forest, Food safety, Food toxicology, Rare earths, REE, Wild food

## Abstract

There is a dearth of data on rare earth elements (REE), yttrium and scandium in foods which extends also to baseline datasets for edible wild mushrooms, though this has started to change in the last decade. Concentrations and shale normalized patterns of REE and Y (REY) were studied by using inductively coupled plasma–quadrupole mass spectrometer in 22 pools (2235 specimens) of *Cantharellus cibarius* (Golden Chanterelle) collected in Poland and also a pool of *C. minor* (Small Chanterelle) (153 specimens) from Yunnan (Chinese Province). The total REY plus Sc varied in *C. cibarius* from 10 to 593 µg kg^−1^ dw whereas that for the Yunnan’s *C. minor* was 2072 µg kg^−1^ dw. *C. minor* from Yunnan has higher REY and Sc compared to the *C. cibarius*. Sc concentrations in twenty *C. cibarius* pools were below 1 µg kg^−1^ dw, but 17 and 27 µg kg^−1^ dw were detected at the other two sites and 66 µg kg^−1^ dw was detected in *C. minor*. The median Y content of *C. cibarius* and *C. minor* was 22 µg kg^−1^ dw and 200 µg kg^−1^ dw. The difference in REY and Sc concentrations and shale normalized patterns between mushrooms from Poland and Yunnan seems to reflect the regional difference in concentration and composition of these elements in the soil bedrock.

**Supplementary Information:**

The online version contains supplementary material available at 10.1007/s11356-023-25210-6.

## Introduction

Rare earth elements (REE) are elements from the group of lanthanides (La, Ce, Pr, Nd, Sm, Eu, Gd, Tb, Dy, Ho, Er, Tm, Yb, and Lu), including scandium (Sc) and yttrium (Y) that are precious metals and are increasingly used in the manufacture of electrical and electronic devices and other developing technologies for industrial, home, and personal devices (Migaszewski and Gałuszka 2015; Migaszewski et al. 2016; Balaram 2019). Estimated mean concentration of REE in the earth’s crust ranges from about 130 to 240 mg kg^−1^, which is in fact much higher than other commonly mined elements and much higher than their corresponding abundances in chondrites (Zepf 2013; Balaram 2019). REE and other elements have become an emerging field of investigation in environmental and food sciences (Aruguete et al. 1998; Migaszewski and Gałuszka 2015; Pagano et al. 2019; Zabowski et al. 1990).

The increasing use of the REE as a whole or in various individual REE applications in the economy can be considered possible sources of REE in forest soils and so also of their accumulation in wild mushrooms. Coal fly ash contains a certain amount of REE (Franus et al. 2015) and as an anthropogenic waste it can be considered a diffusive source of REE deposition in the ground, although it may generally be a poor source. Sewage sludge from wastewater treatment plants can contain REE and especially if such a facility receives input from specific industrial sources that could contain REE (Kaegi et al. 2021). Treated municipal sewage sludge can possibly be used in forested lands (Zabowski et al. 1990) and/or in agricultural soils (under certain circumstances and regulations as seen in Poland) and this could be a source of REE for forest and field mushrooms. Also, phosphorous fertilizers contain some quantities of REE (Volk et al. 1990). Nevertheless, there is lack of data on the effect of using sewage sludge or phosphorous fertilizers (containing REE) in agriculture and the occurrence of REE in mushrooms grown in treated forests or agricultural soils or from cultivars.

Knowledge of the presence of REE in food, including edible mushrooms (fungi), is limited due to trace to ultra-trace levels, lack of robust analytical methods in the past, reliability of published data and lack of information on the risk of REE as potential food contaminants (Balaram 2019; Borovička et al. 2011; Falandysz et al. 2001; Falandysz 2023a, b, c; Grawunder and Gube 2018; Stijve et al. 2004; Zocher et al. 2018). The first data on the occurrence of 14 REE in mushrooms, including species: *Armillaria solidipes* (*A. ostoyae*, Armillaria Root Rot), *Boletus edulis* (King Bolete), *Laccaria amethystina* (Amethyst Deceiver), *Suillus bovinus* (Jersey Cow Mushroom or Bovine Bolete), *Suillus luteus* (Slippery Jack), *Tricholoma equestre* (*T. flavovirens*, Yellow Knight, Man on Horseback or Saddle-shaped Tricholoma), were published in 2001 (Falandysz et al. 2001). As determined by double focusing sector field inductively coupled plasma mass spectrometry, the individual REE were found to be present in these mushrooms in the range from 0.60 µg kg^−1^ dry weight (dw) for Eu to 400 µg kg^−1^ dw for Ce while *A. solidipes* showed relatively higher concentrations of Ce, Nd and La than the other mushroom species (Falandysz et al. 2001). Scandium (Sc) and yttrium (Y) are also considered by some authors along with the REE, but their occurrence in mushrooms was not discussed together with the other lanthanides in earlier studies (Borovička et al. 2011; Falandysz et al. 2001; Grawunder and Gube 2018; Stijve et al. 2002, 2004; Zocher et al. 2018). Soil REE, Y, and Sc levels are a driver of their accumulation in vegetation, including mushrooms (Dołęgowska and Migaszewski 2013; Ichihashi et al. 1992; Markert and Li 1991; Mędyk and Falandysz, 2022; Zocher et al. 2018).

The mycelium absorbs various elements, including REE and toxic elements (e.g., As, Cd, and Hg) from soil and other substrates (with varying absorption rates) and then translocates these to fruiting bodies or the sclerotia (Andersson et al. 2018; Saba et al. 2020; Tyler 1982; Yoshida and Muramatsu 1997; Zhang et al. 2022). Bioconcentration factor (BCF) is a parameter used to quantify the potential of organism to bioconcentrate mineral constituents in abiotic relationships, e.g., macromycete–soil. The BCF is expressed as the quotient of the concentration of an element in the fruiting body to the level in the substrate (e.g., soil) on dry to dry weight basis. BCF values of La and Ce in mushrooms such as *Amanita pantherina* (common name Panthercap mushroom or False Blusher), *Lactarius hatsudake* (Hatsu Take), *Russula mariae* (Purple-bloom), *Suillus granulatus* (Dotted-stem Bolete, Granulated Bolete or Ringless Slippery Jack) and *T. equestre* have been reported to be well below 1, i.e., they showed on bio-exclusion of REE by fungi, with reported quotients ranging from 0.003 to 0.027 for La and from 0.0003 to 0.025 for Ce (Tyler 1982; Yoshida and Muramatsu 1997).

Knowledge of the occurrence and possible role or not of REE in the physiology of macromycetes is negligible so far. Nevertheless, macromycetes accumulate some amounts of REE in fruiting bodies. Generating reliable baseline data sets on the mineral constituent concentration of raw mushrooms from wild biodiversity is the first step in assessing possible dietary intakes and likely health effects. However, other factors to consider when assessing actual consumption include the effect of culinary processing and preservation, and the accessibility/bioavailability of the minerals after ingestion — the release of minerals from the mushroom meal, and intestinal absorption and bioactivity. Venturella et al. (2014) investigated 13 lanthanides and reported the values of Eu Tb, Dy, Ho, Er Tm, Yb, and Lu, below the detection limit, i.e., from 1 to 2 µg kg^−1^ dw. The study also showed no accumulation factor for Eu in *Suillellus queletii* (Deceiving Bolete) (earlier name *Boletus queletii*) and *Leccinellum lepidum* (Neat Bolete) as well as Er in *B*. *queletii* and *L. lepidum*, and Yb in *B*. *queletii* and *L*. *lepidum*. It was also observed that the f-block lanthanides (Ce, Pr, Nd, Sm, and Gd) do not have a bioconcentration factor, except for Sm, which has a value that is below the detection limit for *Rubroboletus satanas* (Devil’s Bolete) (earlier name *Boletus satanas*) (Venturella et al. 2014).

*Cantharellus cibarius* (Golden Chanterelle, Common Chanterelle, Girolle) is a popular species in Europe and fresh mushrooms a very popular seasonal wild food product there as well as in the Northern Hemisphere. That species has been widely characterised for its macro- and micro-nutrient levels (e.g., K, P, S, Mg, Mn, Na, Ca, Cu, Zn, Co and Se) and radiotoxic ^137^Cs (Bakaytis et al. 2021; Drewnowska and Falandysz 2015; Drewnowska et al. 2017; Falandysz and Drewnowska 2015; Falandysz et al. 2016; Mędyk et al. 2017; Mirończuk-Chodakowska et al. 2019; Mleczek et al. 2013). *Cantharellus minor* is a mycorrhizal, edible species, much smaller than the average *C. cibarius*, but has more slender proportions (Kuo 2006). No data on mineral constituents in *C. minor* could be found in the available scientific literature.

There is a dearth of data on the REE content of mushrooms, especially where credible, validated analytical methods were used. The ambiguity of some published REE datasets for mushrooms has been raised clearly (sample cross contamination with soil/sand particles, lack of method sensitivity and resolution, poor limit of detection, poor choice of instrument, spectral interferences) (Borovička et al. 2011; Stijve et al. 2004; Zocher et al. 2018). This derives more from the very low concentrations at which these elements occur compared to some reported values as well as the analytical chemistry approach adopted (methodology, materials and instrumentation or external contamination) (Borovička et al. 2011; Stijve et al. 2004; Zocher et al. 2018). However, the baseline rules when determining REE in mushrooms is the adoption of adequate sampling in terms of the quantity of the fruiting bodies examined (individual sample or preferably as composite samples), sample preparation approach (clean-up from soil particles and risk of particle incrustation within a fruiting body) and the proper choice of analytical method/instrumentation, including use of argon plasma gas in mass spectrometry and the elimination of interferences (Balaram 2019; Bau et al. 2018; Prohaska et al. 1999; Stijve et al. 2004; Zawisza et al. 2011). Golden chanterelles are a very popular seasonal, organic food product across Europe, and this study aimed to characterize the presence of REE in a relatively large sample of this mushroom from Poland. A pooled sample of *C. minor* collected at Yunnan is also included.

## Materials and methods

### Mushrooms — collection and preparation

**Fig. 1:**
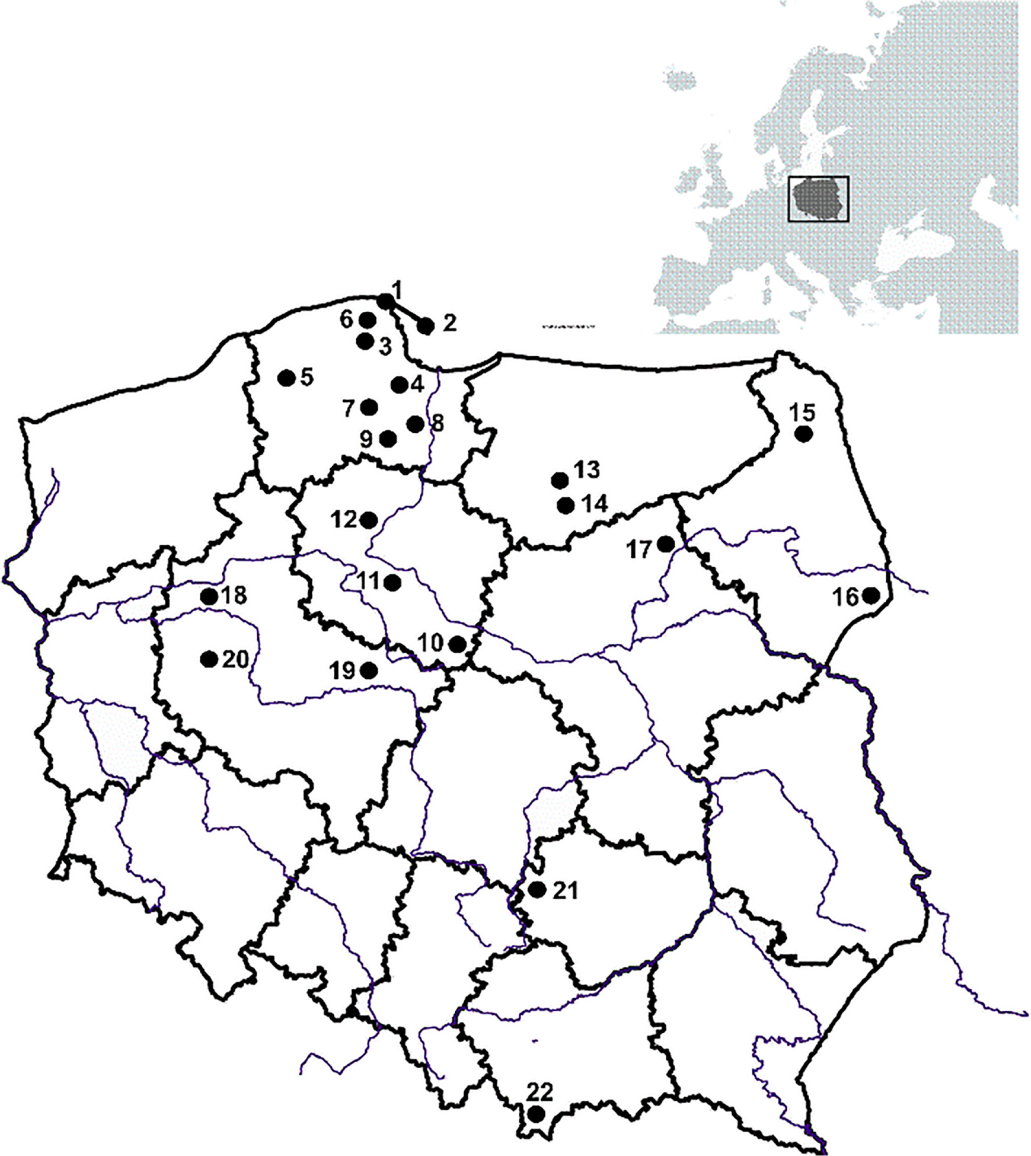
Locations of *C. cibarius* sampling sites in Poland (see Table 2 for id and names of the sites)

Specimens (*n* = 2235) of *C. cibarius* were pooled into 22 composites corresponding to 22 locations across Poland and collected between July–September of 1998–2008. Poland has a moderate and changing climate and landscape flat with Świetokrzyskie, Sudety, Tatra, and Carpathian Mts in the south, and agricultural and forested land dominate. Samples of *C. minor* (153 specimens pooled into 1 composite sample) were collected from the Caoba site in Yuxi Prefecture in Yunnan (China) in July 2013. The sampled sites and their collection identifiers in Poland are presented in Fig. 1 and Table 1S (Supplementary material). *Cantharellus cibarius* mushrooms studied were exclusively from Poland. However, due to reports of elevated levels of REE in soils from Yunnan, a pool of *C. minor* from this region was also investigated. Although *C. minor* is a different species and does not occur in Europe, it is considered a unique material that could function as a positive control. Fresh fruiting bodies (from 4 to 309 per pool) were thoroughly cleaned of impurities, dehydrated at 65 °C to constant weight (dehydrator model MSG-01; MPM Product, Milanówek, Poland, and Ultra FD1000, Ezidri, Australia), hand-ground in a ceramic mortar and stored in clean, airtight polyethylene bags under dry conditions until chemical analysis.

### Elemental analysis

Wet digestion was carried out on aliquots (0.5 ± 0.01 g) of dried and powdered mushroom samples with 8 mL solution of concentrated nitric acid (65%; Suprapur®) in a 1:1 ratio with deionized water and with the addition of 1 mL of hydrogen peroxide solution (30%, Suprapur®) in a high-pressure closed-vessel with the aid of Multiwave 3000 microwave-assisted mineralizer (Anton Paar). After digestion, the solution was quantitatively transferred to centrifuge tubes and made up to 25 mL with distilled water. The digests were stored at 4 °C until the analyses was performed. Analyses was performed by using inductively coupled plasma–quadrupole mass spectrometer (ICP-MS) model Elan DRCII model, PerkinElmer (Table 2S; Supplementary material). Before starting the analyses, the sensitivity of the spectrometer, the background value for mass 220, and the number of counts per second for two positive ions and oxides were checked in each measurement series, using the Elan DRC Setup/Stab/Masscal solution (PerkinElmer) containing 10 µg L^−1^ of Ba, and 1 µg L^−1^ each of Cd, Ce, Cu, In, Pb, Mg, Rh, and U in 0.5% solution of nitric acid (Dołęgowska and Migaszewski 2013; Migaszewski et al. 2016).

Working standard solutions as well as the Rh and Ir internal standards were prepared by the volumetric method on the day of analysis. Internal standards of 1 mg L^−1^ of Rh and 1 mg L^−1^ of Ir were added to each blank, standards, reference materials, and the samples. The volume of the added solutions of the internal standards was 100 µL of Rh and Ir for each 10 mL of medium analyzed. For the preparation of 1 mg L^−1^ internal standard solutions in 2% nitric acid, Rh and Ir solutions of 1000 mg L^−1^ (PerkinElmer) were used. In order to prepare the standard curve, six working solutions were prepared with the analyte concentrations in the range of 1–100 µg L^−1^. These solutions were prepared from the PerkinElmer standard containing 10 mg L^−1^ each of Ce, Dy, Er, Eu, Gd, Ho, La, Lu, Nd, Pr, Sm, Sc, Tb, Tm, Y, and Yb in 2% solution of nitric acid. The standards and samples were diluted with 2% solution of nitric acid and prepared on the day of the analysis by the gravimetric method using 65% solution of nitric acid and deionized water. The standard curves prepared for each analyte were characterized by high values of linear correlation coefficients in the range > 0.999–1 (Dołęgowska et al. 2013; Dołęgowska and Migaszewski 2013; Migaszewski et al. 2016).

Physical interferences were eliminated by using internal standards and diluting the samples. Spectral interferences were eliminated with the use of correction equations and, if possible, determination of different isotopes of the same element. The following reference materials were used to check the accuracy of the measurements: NIST-1573a Tomato leaves and IC-INCT-PVLT-6 Tobacco leaves. The recovery percentages for the elements whose concentrations were included in the certificate were as follows: La 98% and 107%; Ce 63%, Pr 74%, Nd 103%, Sm 104% and 78%, Eu 112%, Tb 76%, Gd 123%, Yb 55%.

## Results and discussion

### Scandium and yttrium concentrations

The content of Sc was < 1 µg kg^−1^ dw in *C. cibarius* from 20 sites and varied from 17 µg kg^−1^ dw for a composite from the Tatra Mountains (outskirts of the city Zakopane) to 27 µg kg^−1^ for the Tuchola Pinewoods in the Lubichowo forest district. *C. minor* from Yunnan showed a much higher Sc content of 66 µg kg^−1^ dw, compared to *C. cibarius* (Table 1).

**Table 1:** Scandium, yttrium, and 14REE in *C. cibarius* from Poland and *C. minor* from Yunnan in China (µg kg^−1^ dw)

Parameter	Element																
Site (ID, Fig. 1)	Sc	Y	La	Ce	Pr	Nd	Sm	Eu	Gd	Tb	Dy	Ho	Er	Tm	Yb	Lu	ΣREE^*^
*Cantharellus cibarius*																	
Coastal Landscape Park (1)	< 1.0	43	46	92	8.3	34	8.8	1.2	5.3	< 1.0	3.0	< 1.0	3.8	< 1.0	< 1.0	< 1.0	204.9
Hel Peninsula, Hel (2)	< 1.0	30	27	30	2.3	7.8	3.4	< 1.0	4.1	< 1.0	3.2	< 1.0	2.4	< 1.0	< 1.0	< 1.0	83.2
Darżlubska Wilderness (3)	< 1.0	32	27	41	1.7	7.5	2.3	< 1.0	4.0	< 1.0	2.2	< 1.0	2.7	< 1.0	< 1.0	< 1.0	91.4
Kolbudy forests (4)	< 1.0	16	28	58	4.8	15	2.1	< 1.0	1.5	< 1.0	2.2	< 1.0	1.2	< 1.0	< 1.0	< 1.0	115.8
Dębnica Kaszubska (5)	< 1.0	19	13	9.2	< 1.0	< 1.0	1.0	< 1.0	< 1.0	< 1.0	1.2	< 1.0	1.2	< 1.0	< 1.0	< 1.0	30.1
Kaszubski Landscape Park (6)	< 1.0	34	59	110	11	29	6.1	< 1.0	3.5	1.0	4.0	< 1.0	3.7	< 1.0	< 1.0	< 1.0	229.8
Wdzydze Landscape Park (7)	< 1.0	20	21	29	2.3	9.4	3.1	< 1.0	3.8	< 1.0	< 1.0	< 1.0	2.5	< 1.0	< 1.0	< 1.0	74.6
Tuchola Pinewoods, Ocypel (8)	< 1.0	17	28	36	2.3	1.2	1.9	< 1.0	< 1.0	< 1.0	< 1.0	< 1.0	1.4	< 1.0	< 1.0	< 1.0	74.8
Tuchola Pinewoods, Lubichowo (9)	27	83	110	220	22	85	17	4.1	18	< 1.0	12	1.3	8.4	< 1.0	5.5	< 1.0	504.8
Gostynińsko-Włocławskie Landscape Park (10)	< 1.0	39	42	52	5.2	9.5	5.3	< 1.0	4.6	< 1.0	2.6	< 1.0	2.3	< 1.0	1.9	< 1.0	127.9
Kujawy, Ciechocinek outskrits (11)	< 1.0	17	1.6	< 1.0	< 1.0	< 1.0	< 1.0	< 1.0	1.3	< 1.0	< 1.0	< 1.0	2.1	< 1.0	< 1.0	< 1.0	10.5
Kujawy, Tuszynki outskrits (12)	< 1.0	31	38	58	4.0	15	2.4	< 1.0	1.8	< 1.0	< 1.0	< 1.0	3.0	< 1.0	< 1.0	< 1.0	125.7
Warmia, Olsztynek outskrits (13)	< 1.0	22	19	23	< 1.0	< 1.0	< 1.0	< 1.0	1.1	< 1.0	< 1.0	< 1.0	2.5	< 1.0	< 1.0	< 1.0	50.6
Warmia, Orzechowo/Olsztynek outskrits (14)	< 1.0	24	22	23	< 1.0	11	5.1	< 1.0	< 1.0	< 1.0	1.9	< 1.0	2.1	< 1.0	< 1.0	< 1.0	69.1
Augustowska Primeval Forest (15)	< 1.0	56	65	115	10	36	10	2.0	5.6	1.1	6.1	< 1.0	5.9	< 1.0	2.6	< 1.0	260.8
Białowieża Primeval Forest (16)	< 1.0	33	26	35	2.1	2.2	3.5	1.3	1.6	< 1.0	3.0	< 1.0	2.5	< 1.0	< 1.0	< 1.0	79.7
Mazowsze, Olszewo-Borki, Commune Lelis (17)	< 1.0	24	17	18	1.4	< 1.0	2.8	< 1.0	2.7	< 1.0	1.2	< 1.0	3.3	< 1.0	1.9	< 1.0	51.3
Notecka Forest (18)	< 1.0	19	27	27	3.0	< 1.0	1.3	< 1.0	1.8	< 1.0	< 1.0	< 1.0	2.7	< 1.0	< 1.0	< 1.0	66.8
Wielkopolska, Zagórów (19)	< 1.0	28	24	28	< 1.0	9.1	3.1	1.0	1.2	< 1.0	< 1.0	< 1.0	2.3	< 1.0	< 1.0	< 1.0	72.2
Wielkopolska, Porażyn (20)	< 1.0	18	19	19	< 1.0	1.6	< 1.0	< 1.0	< 1.0	< 1.0	< 1.0	< 1.0	2.3	< 1.0	< 1.0	< 1.0	46.9
Świetokrzyskie region, Włoszowa (21)	< 1.0	27	57	94	9.4	29	4.6	1.1	3.6	< 1.0	2.4	< 1.0	2.6	< 1.0	< 1.0	< 1.0	206.2
Tatra Mountains, Zakopane (22)	17	83	140	250	27	96	20	3.4	19	8.3	14	1.4	7.9	< 1.0	4.8	< 1.0	592.8
Mean	< 1.0	32	39	62	5.4	18	4.8	0.98	3.9	0.9	2.9	0.58	3.1	0.5	1.1	0.5	143.7
SD	WD	19	32	64	7.0	26	5.1	0.98	5.0	1.7	3.6	0.25	1.9	WD	1.4	WD	147.8
Median	< 1.0	22	27	35	2.3	9.2	3.1	0.5	2.2	0.5	2.0	0.5	2.5	0.5	0.5	0.5	77.2
Range — minimum	< 1.0	16	1.6	< 1.0	< 1.0	< 1.0	< 1.0	< 1.0	< 1.0	< 1.0	< 1.0	< 1.0	1.2	< 1.0	< 1.0	< 1.0	10.5
Range — maximum	27	83	140	250	27	96	20	4.1	19	8.3	14	1.4	8.4	< 1.0	5.5	< 1.0	592.8
*Cantharellus minor*																	
China, Yunnan	66	200	480	940	95	360	58	9.6	56	2.5	34	5.7	18	1.6	11	< 1.0	2071.9

ID of the sampling site; for localization, see in Fig. 1; ^*^data without rounding(If a result was < 1.0 µg kg^-1^ dw, the value of 0.5 µg kg ^-1^ dw was used to calculate ΣREEs); *WD*, without data

In the earliest study of Sc in mushrooms (using instrumental neutron activation analysis, INAA), the element was detected in *Lycoperdon pyriformis* (Pear-shaped Puffball) at 480 µg kg^−1^ dw and in *Scleroderma verucosa* (Scaly Earthball) at 1300 µg kg^−1^ dw (Horowitz et al. 1974) — both results are substantially higher compared to the *Cantharellus* spp. investigated in this study (Table 1). INAA was used to determine Sc in a series of 115 individual mushrooms and the overall concentrations were in the range of 2 to 240 µg kg^−1^ dw (Řanda and Kučera 2004). For different species, this varied from 2.5 ± 0.3 µg kg^−1^ dw for *Lycoperdon perlatum* (also called Common Puffball, Gem-studded Puffball or Devil’s Snuffbox) to 76 ± 2 µg kg^−1^ dw for *C. cibarius*. In *Cantharellus lutescens* (Yellow Foot) and *Cantharellus pallens* (Pale Chanterelle) collected from the Bohemia (Czechia), the Sc contents were 44 ± 8 and 32 ± 1 µg kg^−1^ dw, respectively (Řanda and Kučera 2004). Saprotrophic *Macrolepiota procera* (field parasol or parasol mushroom) collected across Poland contained 28 ± 48 µg kg^−1^ dw (total < 1–160 µg kg^−1^ dw) Sc in the caps (determined using the Quadruple inductively coupled argon plasma–mass spectrometry analysis) and 28 ± 25 µg kg^−1^ dw (total 5.3–55 µg kg^−1^ dw) in the whole fruiting bodies (Falandysz et al. 2017).

In a more recent study, Sc was found at relatively higher concentrations in soil from the regions of Serbia (total range of 2000 to 13,000 µg kg^−1^ dw) but much lower concentrations were observed in *M. procera* from the same location, with mean values ranging from 14 ± 16 to 110 ± 70 to µg kg^−1^ dw (range < LOD to 240 µg kg^−1^ dw for caps, while in the stipes, concentrations ranged from 63 ± 43 to 80 ± 62 µg kg^−1^ dw) (Vukojević et al. 2019). Vukojević et al. demonstrated that Sc occurs minimally in *M. procera* compared to the soil substrate (where the mycelium grows). The BCF values varied from 0.013 to 0.017, showing bio-exclusion of the element in matured fruiting bodies of this species (Tyler 1982; Vukojević et al. 2019).

Yttrium was detected in all the composite samples of *C. cibarius* (median value of 22 µg kg^−1^ dw) and also in the *C. minor* collect from Yunnan (200 µg kg^−1^ dw) (Table 1). Similarly, as was observed for Sc, the *C. cibarius* originating from the outskirts of Zakopane and also from the Tuchola Pinewoods showed greater concentrations of Y (both values were 83 µg kg^−1^ dw). The Y concentrations for samples from sites at the Coastal Landscape Park and Augustowska Primeval Forest are 43 and 56 µg kg^−1^ dw, indicating more accumulation, far above the overall median value of 22 µg kg^−1^ dw (Table 1). Higher Sc and REY were observed in the *C. minor* compared to the *C. cibarius* (mostly by an order of magnitude), thereby confirming literature reports that these elements are higher in the bedrock of Yunnan and that the amounts translocated to the fruiting bodies also depends to a great extent on the amount in the topsoil substrate.

Compared to previous studies of Y in *M. procera*, a reported concentration of 74 ± 39 µg kg^−1^ dw in the caps and 110 ± 30 µg kg^−1^ dw in the whole mushrooms, indicated higher retention in the stipe and much slower translocation via the stipe to the cap (Falandysz et al. 2017). In a study of *M. procera* from Serbia, the element Y was found to be in the range of 9 ± 8 to 30 ± 27 µg kg^−1^ dw in the caps and, from 45 ± 39 to 74 ± 140 µg kg^−1^ dw in the stipes, with low BCFs of 0.009, indicating that this could have derived from the high levels of Y in the soil (3600 to 12,000 µg kg^−1^ dw; rounded) (Vukojević et al. 2019). The earliest study of Y in mushrooms (in *Albatrellus pes-caprae*; current name *Scutiger pes-caprae*, Goat’s Foot) in 2002 reported some results that were relatively elevated (range from < 50 to 2000 µg kg^−1^ dw; median of 270 µg kg^−1^ dw) (Stijve et al. 2002). It was later clarified that this was because of the difficulty in avoiding contamination with sand/soil particles during analysis (Stijve et al. 2004). A specimen of *B. edulis* from Germany was shown to contain about 18 µg kg^−1^ dw of Y, while a study of 10 composite samples (201 fruiting bodies) of the same species from Poland reported a concentration of 62 ± 76 µg kg^−1^ dw (Bau et al. 2018; Falandysz et al. 2022). Borovička et al. (2011) studied REE in 36 species of ectomycorrhizal (26 samples) and saprobic (25 samples) macro-fungi from unpolluted sites with differing bedrock geochemistry and reported concentrations that did not exceed 360 µg kg^−1^ dw. They also observed that their distribution more or less followed the trend observed in post-Archean shales and loess.

### Concentration of 14 REE

The concentrations (µg kg^−1^ dw) of the 14 REE for the samples collected from locations in Poland varied widely ranging from < 1.0 to 250 (median of 35) for Ce; < 1.0 to 27 (2.3) for Pr; < 1.0 to 96 (9.2) for Nd; < 1.0 to 20 (3.1) for Sm; < 1.0 to 4.1 (0.5) for Eu; < 1.0 to 19 (2.2) for Gd; < 1.0 to 8.3 (0.5) for Tb; < 1.0 to 14 (2.0) for Dy; 1.0 to 1.4 (0.5) for Ho; 1.2 to 8.4 (2.5) for Er; < 1.0 to < 1.0 (0.5) for Tm; < 1.0 to 5.5 (0.5) for Yb; and < 1.0 to < 1.0 (0.5) for Lu. For the sample collected from Yunnan, the corresponding values were 480 for La, 35 for Ce, 2.3 for Pr, 9.2 for Nd, 3.1 for Sm, 0.5 for Eu, 56 for Gd, 2.5 for Tb, 34 for Dy, 5.7 Ho, 18 for Er, 1.6 for Tm, 11 for Yb, and < 1.0 for Lu. The sum of the 14 REE concentrations (ΣREE) which includes Sc and Y for the various locations investigated in Poland varied from 10.5 µg kg^−1^ dw for the Ciechocinek in Kujawy region to 592.8 µg kg^−1^ dw for Tatra Mountains, Zakopane, whereas that for the Yunnan sample was 2071.9 µg kg^−1^ dw (Table 1). Gałuszka et al. (2020) studied REE in plants collected in areas impacted by acid mine drainage in Poland and reported that sum of REE ranged from 0.069 to 28 mg kg^−1^ dw for one site and from 0.36 to 26.4 mg kg^−1^ dw for another. Studies have shown that moss accumulates more REE than plants. Gałuszka et al. (2020) observed REE for mosses that was 11 times higher than that for vascular plants. This may depend on unique features of mosses — they are non-vascular plants with simple tissues but rootless and featured by high surface-to-volume ratio, slow growth rate, aerial uptake of nutrients, and high ion-exchange capacity allowing them to bioconcentrate some atmospheric pollutants over long periods of time.

Concentrations of REE well above the median value were noted for locations such as Coastal Landscape Park (id 1), with REE of 204.9 µg kg^−1^ dw; Kaszubski Landscape Park (id 6) with 229.8 µg kg^−1^ dw, Tuchola Pinewoods in Lubichowo (id 9) with 504.8 µg kg^−1^ dw; Augustowska Primeval Forest (ID 15) with 260.8 µg kg^−1^ dw; Świetokrzyskie region in Włoszowa (id 21) with 206.2 µg kg^−1^ dw and Tatra Mountains, in Zakopane (id 22) with 592.8 µg kg^−1^ dw (Table 1).

The Baltic Sea marine sands at the southern coastal area are relatively rich in REE and have been reported as containing a “vast preponderance of light REE (LREE; La, Ce, Pr, Nd, Pm, Sm, Eu) among the rare earths” followed by Ce, La, and Nd and lastly by Y (Mikulski et al. 2016). On the other hand, the montane soils of the Świetokrzyskie and Tatra Mountains regions have a rocky background, while the Augustowska Primeval Forest region has deposits of sand and gravel, with some ore anomalies (Bońda et al. 2020). Forest areas of the Coastal Landscape Park, Kashubian Landscape Park and Bory Tucholskie (Lubichowo) have a sandy bedrock with a sandy topsoil, but the soil has not been investigated, and it has not been possible to confirm how the soils are responsible for the relatively higher REE observed in *C. cibarius* from these locations.

**Fig. 2:**
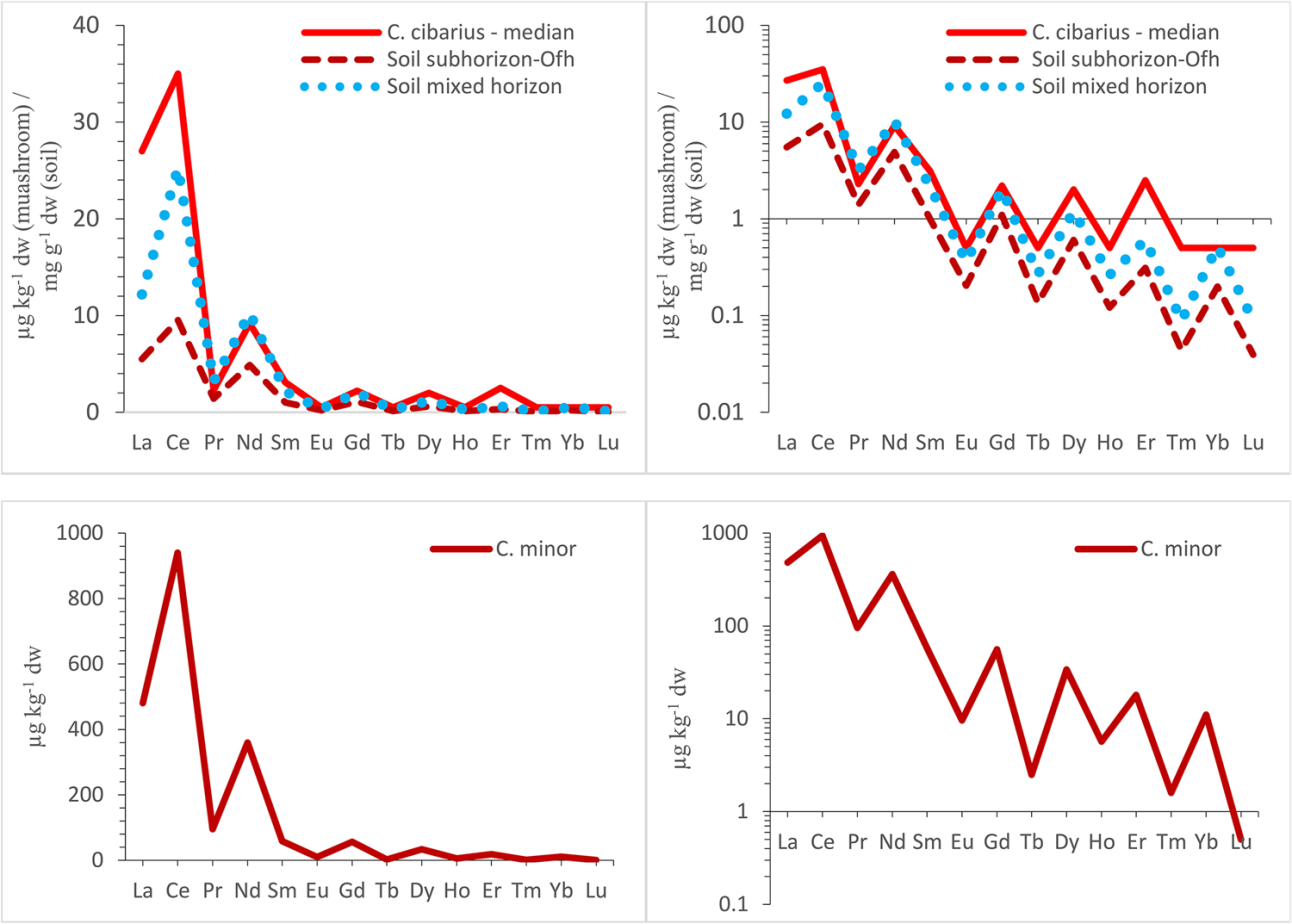
Distribution pattern of REE in *C. cibarius* and topsoil in Poland (data on REE in topsoil adapted from Dołęgowska et al. 2013), and in *C. minor*

Distribution of REE concentrations in *C. cibarius* (median values) and in *C. minor* follows the Oddo-Harkins rule and shows characteristic “zigzag” pattern (Fig. 2). This concentration pattern of REE in *C. cibarius* fits with natural concentration pattern of REE in topsoil in Poland (Fig. 2).

### REE — *Cantharellus* mushrooms — human exposure

Balaram (2019) and Pagano et al. (2019) recently reviewed the available data on human risk from REE due to occupational and environmental exposures. Similarly, Doulgeridou et al. (2020) reviewed the risk from REE in plant-based foods. REE are not considered an essential compound in human nutrition. Lanthanum, which like other REE is known as a seeker for calcium (Ca), has been found to mimic (replace) Ca in some living function of bacteria *Methylobacterium radiotolerans* (Hibi et al. 2011). Because of the low or ultra-low occurrence of REE in foods including edible wild mushrooms, their dietary exposure is negligible. A few studies report data on REE which were considered substantially excessive — and the reasons for this have been discussed by Borovička et al. (2011), Zocher et al. (2018) and Falandysz (2023b).

The elements, Ce, La, and Nd were observed in higher concentrations in *C. cibarius* compared to other REE. Their contributions to the median value of REE concentrations was 42% for Ce, followed by La (35%) and then Nd (12%), and altogether, they accounted for 92% of the REE. The contribution of Ce, La, and Nd to REE is relatively high in specimens of *C. cibarius* from the Zakopane site from Poland (42, 24, and 16% respectively) and this is close to the proportions observed in *C. minor* which originated from a polymetallic soil background of Yunnan (45, 25, and 17%, respectively). China has rather high REE soil concentrations (Liang et al. 2005).

The mean concentration of summed REE plus Sc and Y determined in *C. cibarius* is 176 ± 167 µg kg^−1^ dw (rounded). This value is clearly low compared to values earlier reported for *M. procera* (mean concentration of 500 µg kg^−1^ dw in caps and 750 µg kg^−1^ dw in the whole fruiting bodies) or the whole *B. edulis* from Poland with 430 ± 430 µg kg^−1^ dw (median 310 µg kg^−1^ dw) (Falandysz et al. 2017, 2022). The amounts of the REE (plus Sc and Y) in *C. cibarius* mushrooms can be considered to be rather small (even negligible) from the point of view of food safety experts (food toxicologists and nutritionists). The reason can be that REE have some predilection to calcium (Ca) in organisms (Ascenzi et al. 2020), which is essential for fungi and undergoes homeostatic regulation (Lange and Peiter 2020). REE tend to accumulate in the bone structure (Chen and Zhu 2008). Vukojević et al. (2019) reported a positive correlation between lanthanides and Ca in mushrooms. Fruiting bodies of wild mushrooms are much richer in Ca (containing one to a few hundred mg kg^−1^ dw) than REE (Malinowski et al. 2021). It would be interesting to investigate further the possible relationship between REE and Ca in mushroom fruiting bodies, although at the present stage, the amount of reliable data on REE in mushrooms is too scarce. Calcium contents of *C. cibarius* collected in Poland and elsewhere (Table 2; including some batches of mushrooms in this study) were three orders of magnitude in excess of REE including Sc and Y (Table 1), i.e., in the range (median) from 100 to 270 mg kg^−1^ dw (60 pools of mushroom with 847 fruiting bodies) and from 200 to 520 mg kg^−1^ dw (60 pools of mushroom with 141 fruiting bodies) (Drewnowska and Falandysz 2015; Falandysz and Drewnowska 2015).

**Table 2:** Calcium concentration (mg kg^−1^ dw) in *Cantharellus* spp.

Species	*n*	Year	Ca (rounded values)	Ref.
*Cantharellus cibarius* Common Chanterelle				
Finland	6	1976	360 (330–420)	A
Finland	17	1977–1999	530	B
Poland, northern	3 (30)	1994	340 ± 130 (190–430)	C
Czech Republic	1	p. 2004	350 ± 34	D
Czech Republic	1	p. 2005	350	E
Czech Republic	1	p. 2005	160	E
Poland	6 0(141)	2004–2007	200–520 (150–2300)	F
Poland	60 (847)	1998–2003	210 ± 59–680 ± 620	G
*Cantharellus lutescens* Golden Chanterelle				
Czech Republic	1	p. 2004	240 ± 46	D
Czech Republic	1	p. 2005	240	E
Czech Republic	1	p. 2005	260	E
*Cantharellus pallens*				
Czech Republic	1	p. 2004	500 ± 50	D
Czech Republic	1	p. 2005	500	E
*Cantharellus tubaeformis* Yellow foot				
Finland	17	1977–1999	220	B

*n* (quantity of fruiting bodies or quantity of pooled samples and quantity of fruiting bodies in a pool — in parentheses

*Ref*., references: A (Varo et al. 1980), B (Pelkonen et al. 2008), C (Falandysz et al. 2001), D (Řanda and Kučera 2004), E (Řanda et al. 2005), F (Drewnowska and Falandysz 2015), G (Falandysz and Drewnowska 2015)

### Shale normalized patterns of REE in *Cantharellus* mushrooms

Certain mushrooms were found to be more or less species-specific accumulators of some elements (e.g., Ag, As, Cd, Hg, Se, and V) and show good bioconcentration potential for these elements (Sácký et al. 2014; Komorowicz et al. 2019). No such phenomena could be identified thus far in the case of mushrooms and REE. As mentioned, the BCF values of REE determined for mushrooms were well below 1 and showing on bio-exclusion of REE by these organisms (Yoshida and Muramatsu 1997; Tyler 1982; Zocher et al. 2018; Mędyk and Falandysz 2022). It has been found that vegetation native to the sites impacted by acid mine drainage were good at bioconcentrating REE (Gałuszka and Migaszewski 2018; Gałuszka et al. 2020). No similar evidence could be found up to now in the case of edible wild mushrooms, which typically are collected from the forested areas and woodlands considered to be unpolluted, while mushrooms from anthropogenically impacted sites (metal smelters, metal refineries, legacy mine areas, cities etc.) can be contaminated with typical heavy metals (Zabowski et al. 1990; Árvay et al. 2014; Falandysz 2016, 2017).

As evidenced, ΣREE were found to be higher (i.e., above 200 µg kg^−1^ dw) in *C. cibarius* collected from six of the twenty-two studied locations (Table 1). Also, Y was elevated for these six locations whereas Sc was higher at two of the locations (for other location Sc was < 1 µg kg^−1^ dw) (Table 1). Considering that studies have shown that for mosses and vascular plants, a local abundance and bioavailability of REE in soil are important determinants for bioconcentration in above ground plant parts (Gałuszka and Migaszewski 2018; Gałuszka et al. 2020). It is also possible that the anomaly of REE in mushrooms could reflect both the REE natural occurrence in forest soil due to parent soil bedrock as well as potential releases from environmental pollution (especially if any anomalies in normalized pattern of the REE could be identified). This theoretically can be identified by the constructive analysis of the graphs of their normalized distribution in relation to their occurrence in shales with the aim of detecting and interpreting any hypothetical anomaly. Nevertheless, extensive research has been carried out in recent years and evidence has been found that distribution patterns of REE over Europe are entirely attributable to geology without any evidence of REE anthropogenic pollution (Fedele et al. 2008).

**Fig. 3:**
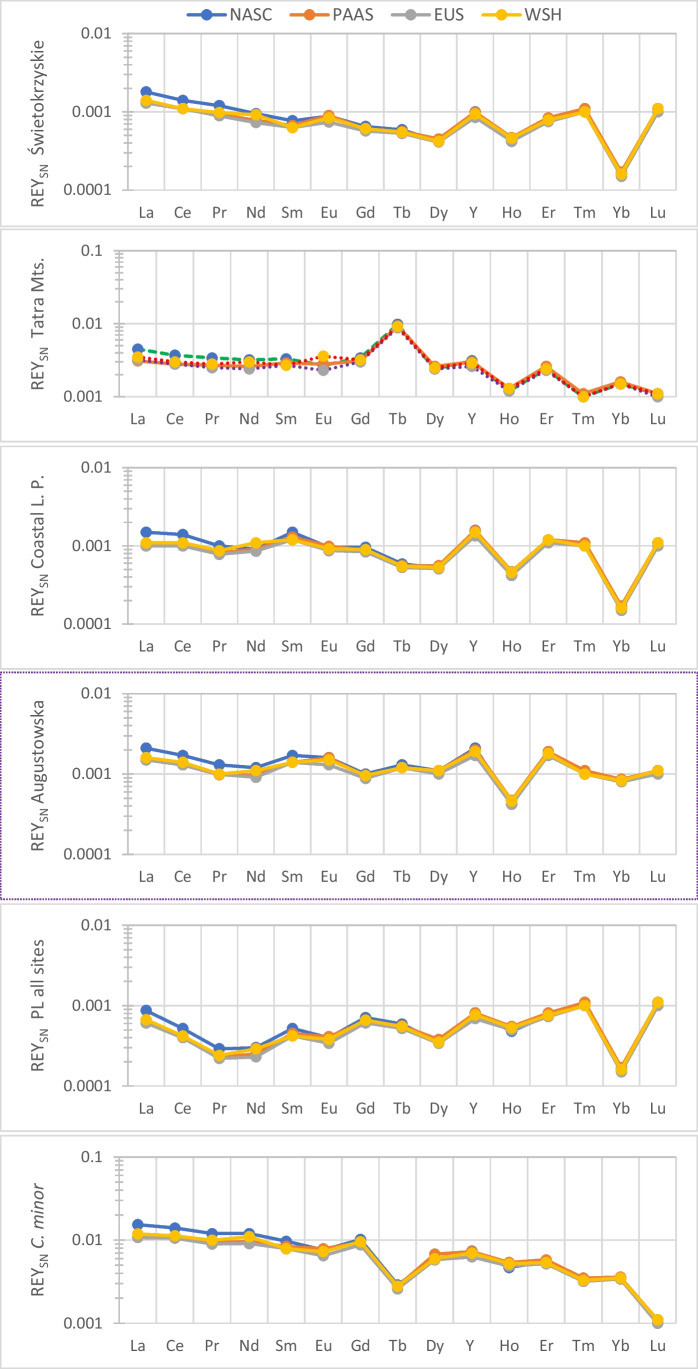
NASC-, PAAS-, EUS- and WSH-shale normalized patterns of REY in *C. cibarius* from the Świetokrzyskie region in Włoszowa, Tatra Mountains in Zakopane, Coastal Landscape Park (Baltic Sea), Augustowska Primeval Forest and from all sites jointly in Poland (based on median concentration values), and in *C. minor* from Yunnan in China

This study also examined the shale-normalized distribution of REY for a few collections of *C. cibarius* and *C. minor* (Fig. 3; REE and REY shale normalized pattern combinations in Figs. 1S-4S, Electronic Supplementary Material). The mean and median values of the REE concentrations in selected *C. cibarius* samples and in *C. minor* were normalized against the North American Shale Composite (NASC) and Post-Archean Australia Shales (PAAS) (Dołęgowska and Migaszewski 2013) as well as European Shale and World Shale (Bau et al. 2018; Migaszewski and Gałuszka 2019) (Fig. 3, Figs. 1S, 2S, 3S, and 4S, Tables 3S, 4S, and 5S, Electronic Supplementary Material; where the concentration was < 0.1 µg kg^−1^ dw a value of 0.5 µg kg^−1^ dw was used, this is roughly the limit of quantification).

The REE are often grouped into light REE (La, Ce, Pr, and Nd; LREE), middle REE (Sm, Eu, Gd, Tb, and Dy; MREE), and the heavy REE (Ho, Er, Tm, Yb, and Lu; HREE). Anomalies were observed in the REY plots among the MREE and more predominantly in HREE (Figs. 1S, 2S, and 3S) for all the sites considered except for the *C minor* from Yunnan China (cf. Figure 4S; the normal and log-normalized plots). For the *C minor* from Yunnan China, anomalies were observed more for Tb and possibly also for Lu. Tb negative anomaly was also observed for the Tuchola Pinewood. Considering normalization of the median values for all sites (Fig. 4Sg’), the highest anomaly (negative) was observed for Yb, similar to the pattern observed for the Świetokrzyskie region in Włoszowa (Fig. 2Sa´), the Baltic Sea Coastal Landscape Park (Fig. 2Sc´) and Kaszubski Landscape Park. Kaszubski Landscape Park (Fig. 2Sd´).

## Conclusion

Median REE concentrations determined in 22 collective samples of *C. cibarius* collected in Poland indicate their negligible content, and thus no risk to consumers of this popular wild mushroom. The pooled sample of *C. minor* from Yunnan was richer in REE than *C. cibarius* from Poland, and this may be a good reason for more extensive research on REE in Yunnan mushrooms.

## Supplementary Information

Below is the link to the electronic supplementary material.

Supplementary file1 (PDF 655 KB)

## Data Availability

Not applicable.
