# Correction to: Scandium, yttrium, and lanthanide occurrence in *Cantharellus cibarius* and *C. minor* mushrooms

**DOI:** 10.1007/s11356-023-25961-2

**Published:** 2023-02-16

**Authors:** Małgorzata Mędyk, Jerzy Falandysz, Innocent Chidi Nnorom

**Affiliations:** 1 Environmental Chemistry & Ecotoxicology, University of Gdańsk, 63 Wita Stwosza Str., 80‑308 Gdańsk, PL Poland; 2 Department of Toxicology, Faculty of Pharmacy, Medical University of Lodz, 1 Muszyńskiego Street, 90‑151 Łódź, Poland; 3 Analytical/Environmental Unit, Department of Pure and Industrial Chemistry, Abia State University, Uturu, Nigeria

**Correction to: Environmental Science and Pollution Research**

10.1007/s11356-023-25210-6

The correct family name of the last Author is Nnorom.

The Original article has been corrected.

