# Supplementary material for: Scandium, yttrium, and lanthanide occurrence in Cantharellus cibarius and C. minor mushrooms
Source: Environ Sci Pollut Res Int. 2023 Jan 12;30(14):41473–84. doi: 10.1007/s11356-023-25210-6 (PMC10067650; doi:10.1007/s11356-023-25210-6)
Supplement: Supplementary file 1 — Supplementary file1 (PDF 655 KB) [file 11356_2023_25210_MOESM1_ESM.pdf]

Electronic Supplementary Material

Scandium, yttrium and lanthanoids in *Cantharellus cibarius* and *C. minor* mushrooms

Małgorzata Mędyk[1*], Jerzy Falandysz[1#], Nnorom Innocent Chidi[2]

[1]Medical University of Łódź, Faculty of Pharmacy, Department of Toxicology, 1 Muszyńskiego Street, 90-151 Łódź, Poland

[2]Analytical / Environmental Unit, Department of Pure and Industrial Chemistry, Abia State University, Uturu, Nigeria

[#]Corresponding author

[*]PhD Student

Table 1S. *Cantharellus* spp. sampling details (sampling site ID's, site names and location, year of collection and sample size)

Site ID (see Fig. 1)	Species and site names	Year of collection	Quantity of fruiting bodies in a pool
	Cantharellus cibarius		
1	Pomerania, Seacoast Landscape Park	2007	120
2	Pomerania, Hel Peninsula, Hel outskrits	2004	53
3	Pomerania, Darżlubska Wilderness, Wejherowo	2000	90
4	Pomerania, Kolbudy forests	2006	32
5	Pomerania, Dębnica Kaszubska	2003	160
6	Pomerania, Kaszubski Landscape Park, Borucino	2000	90
7	Pomerania, Wdzydze Landscape Park, Dziemiany	2000	309
8	Pomerania, Tuchola Pinewoods, Pelplin and Ocypel	1999	100
9	Pomerania, Tuchola Pinewoods, Lubichowo	2007	120
10	Kujawy, Gostynińsko-Włocławski Landscape Park, Goreń	2001	81
11	Kujawy, Ciechocinek outskrits	2004	51
12	Kujawy, Tuszynki outskrits	2006	44
13	Warmia, Olsztynek outskrits	2003	160
14	Warmia, Orzechowo/Olsztynek outskrits	2008	167
15	Suwalskie region, Augustowska Primeval Forest	2006	160
16	Podlasie, Białowieża Primeval Forest	1998	160
17	Mazowsze, Olszewo-Borki, Commune Lelis	2007	160
18	Wielkopolska, Notecka Forest, Jesionna	2000	160
19	Wielkopolska, Zagórów	2007	150
20	Wielkopolska, Porażyn	2008	4
21	Świetokrzyskie region, Włoszowa	2007	120
22	Małopolska, Tatra Mountains, Zakopane	2007	71
	Cantharellus minor		
China	China, Yunnan Province, Yuxi, Caoba	2013	153

Table 2S. Operating condition of the ICP-MS

Parameter	Value
Sweep per reading	20
Readings per replicate	4
Replicates	3
Carrier gas flow rate	1.03 L min^{-1}
Make-up gas flow rate	1.20 L min^{-1}
Plasma gas flow rate	15 L min^{-1}
Lens voltage	7.50 V
Analog detector voltage	1650 V
Pulse detector voltage	950 V
Plasma generator power	1275 W
Scan type	Peak hopping
Ions scan time	20 ms

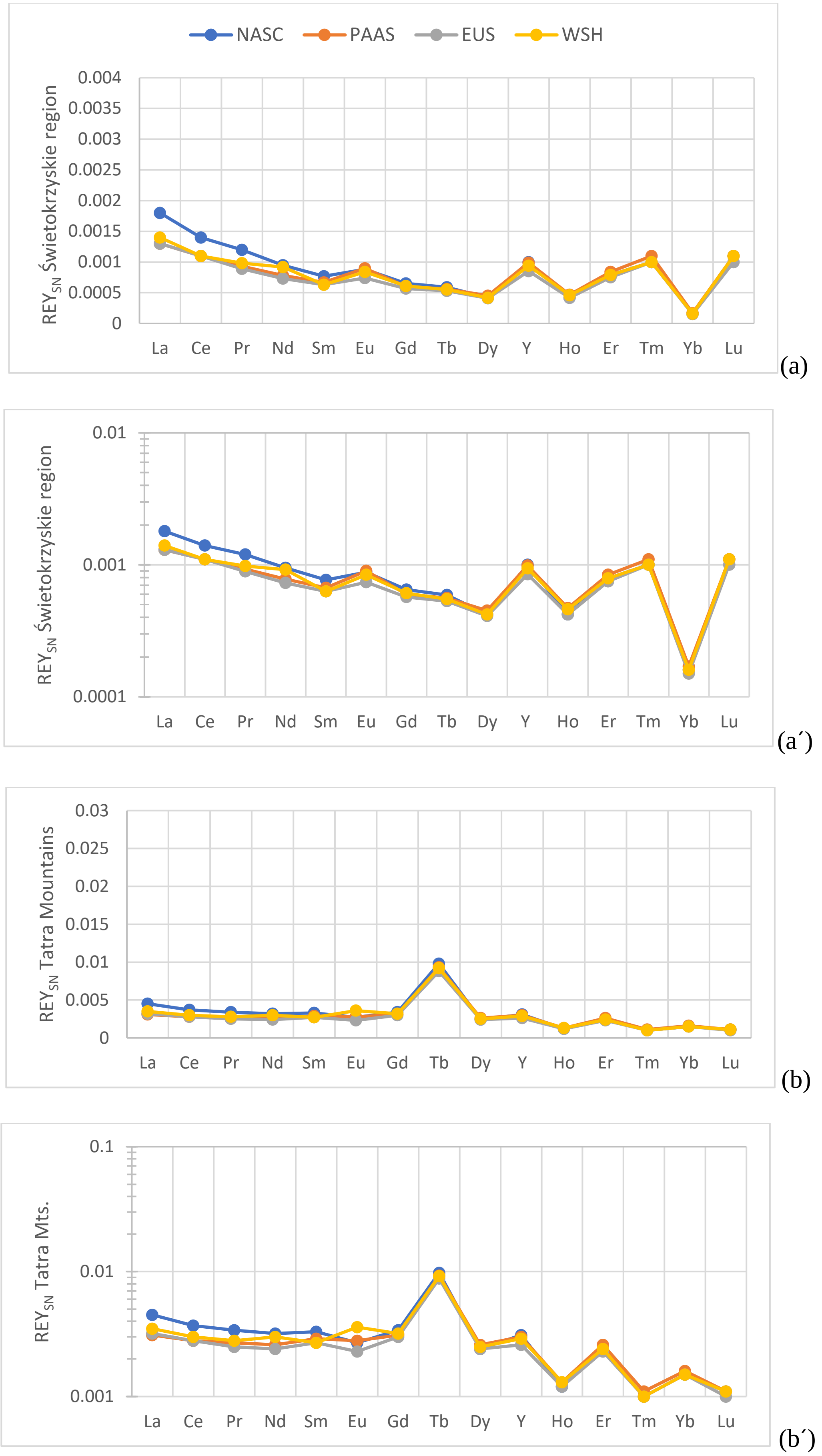

Figure 1S. NASC-, PAAS-, EUS- and WSH- shale normal and log-normalized patterns of REY in *Cantharellus cibarius* from (a & a´) the Świetokrzyskie region in Włoszowa and (b & b´) Tatra Mountains – Zakopane site.

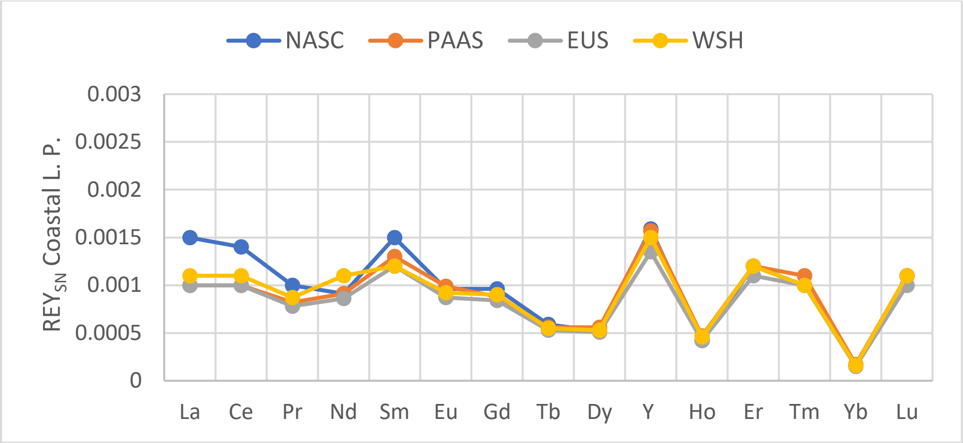

(c)

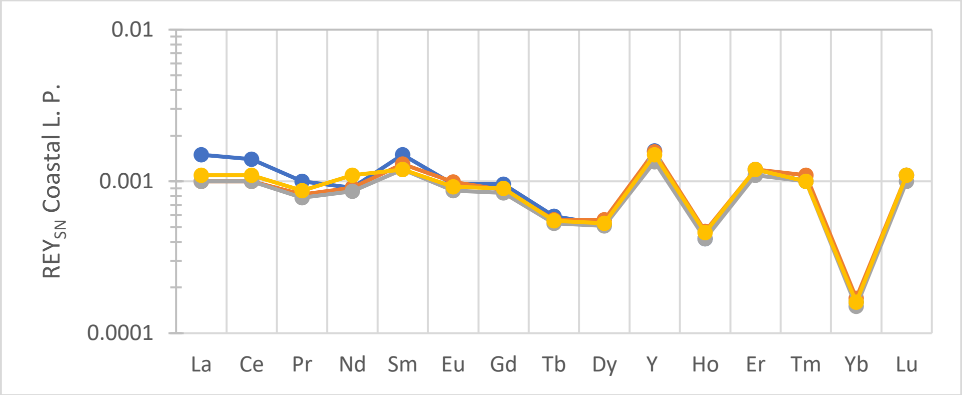

(c′)

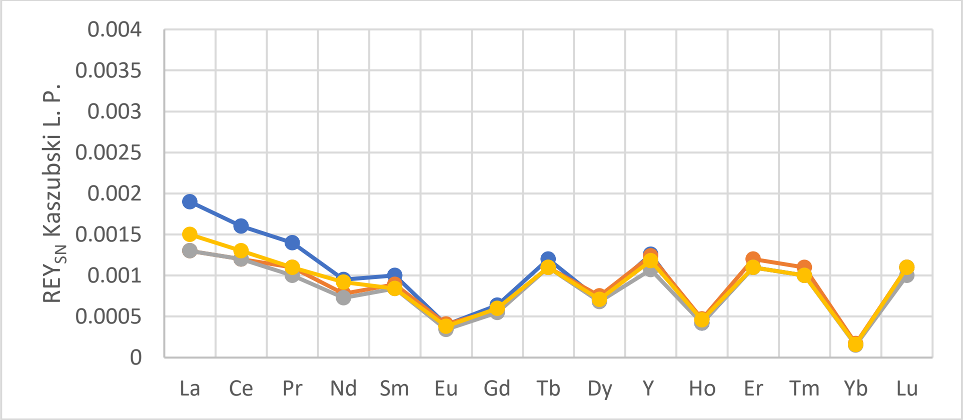

(d)

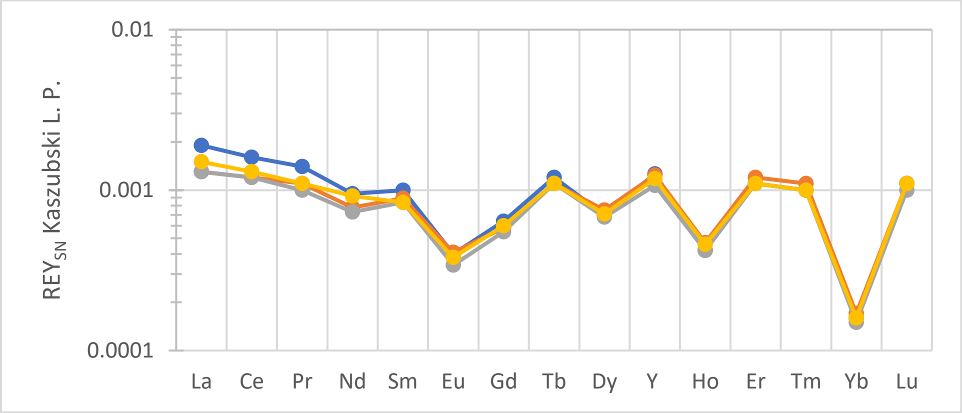

(d′)

Figure 2S. NASC-, PAAS-, EUS- and WSH- shale normal and log-normalized patterns of REY in *Cantharellus cibarius* from (c & c′) the Baltic Sea Coastal Landscape Park and (d & d′) Kaszubski Landscape Park.

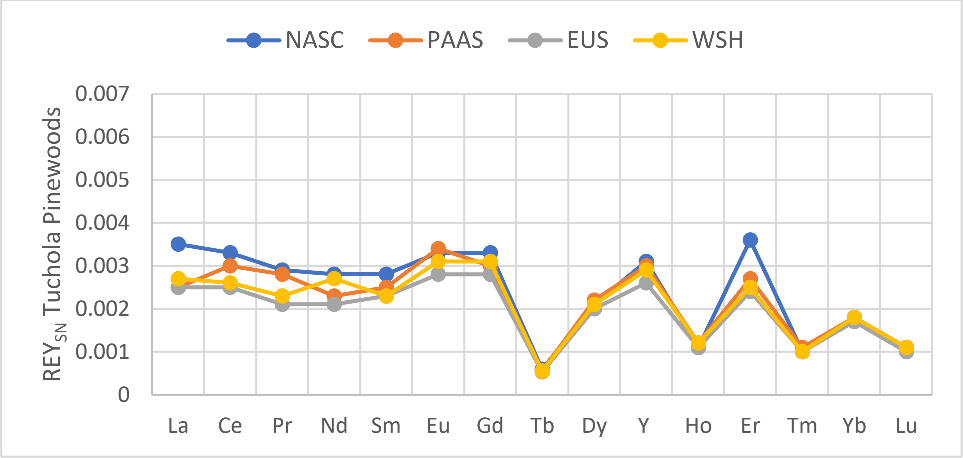

(e)

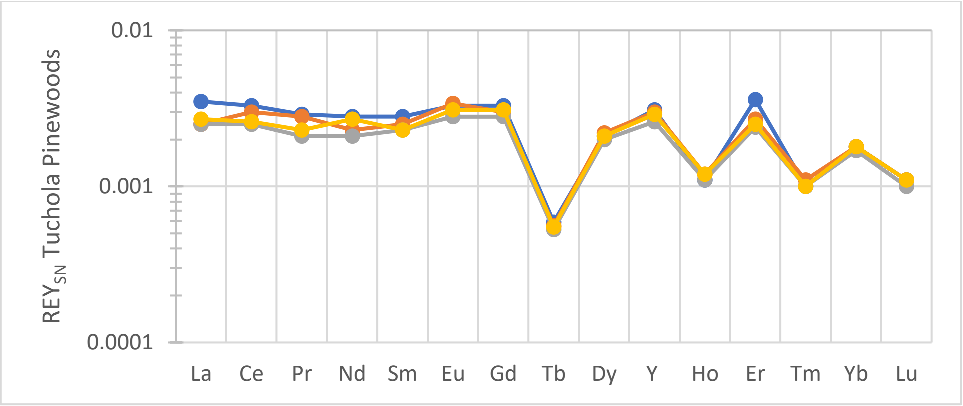

(e´)

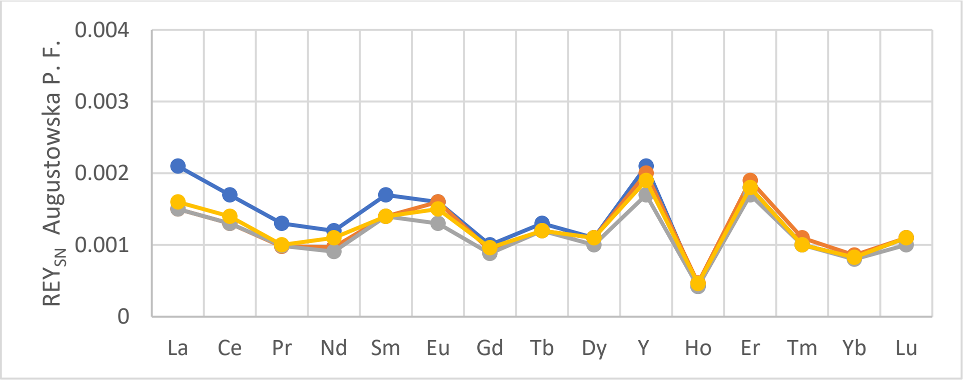

(f)

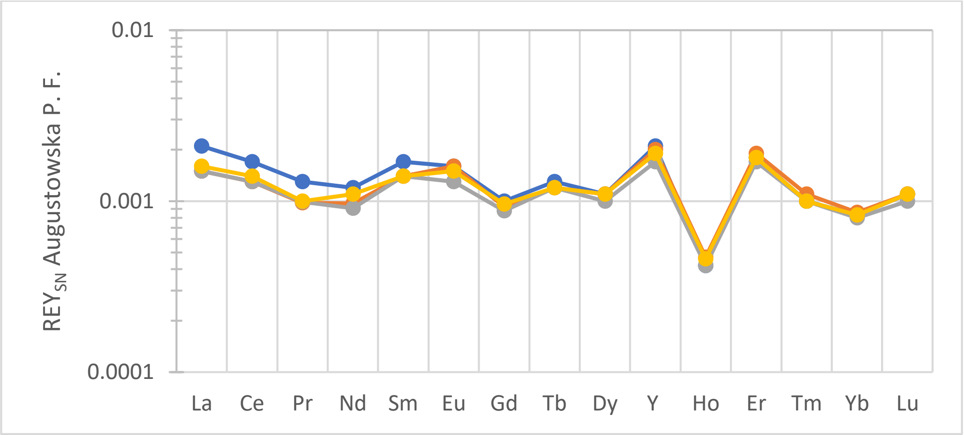

(f´)

Figure 3S. NASC-, PAAS-, EUS- and WSH- shale normal and log-normalized patterns of REY in *Cantharellus cibarius* from the (g & g´) Tuchola Pinewoods in Lubichowo and (h & h´) Augustowska Primeval Forest.

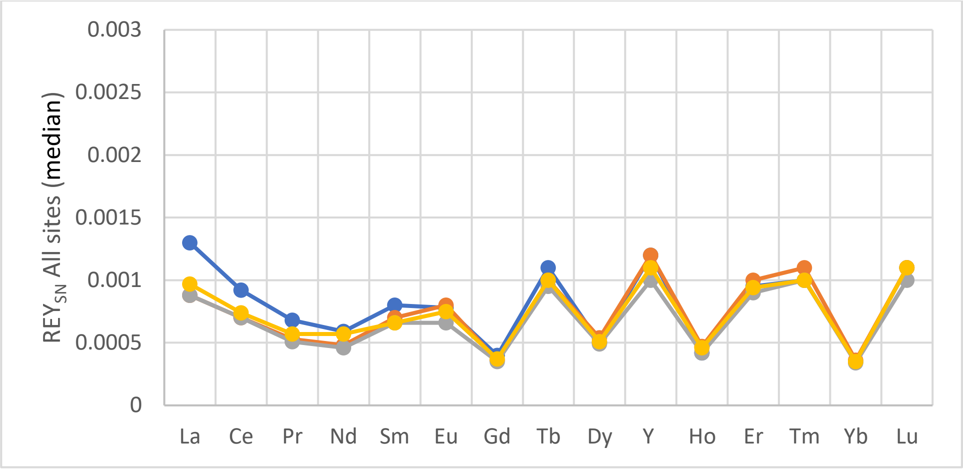

(g)

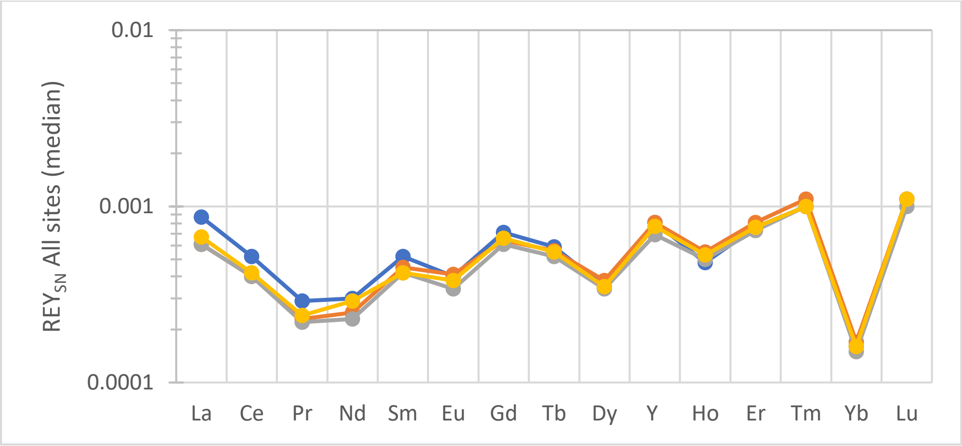

(g´)

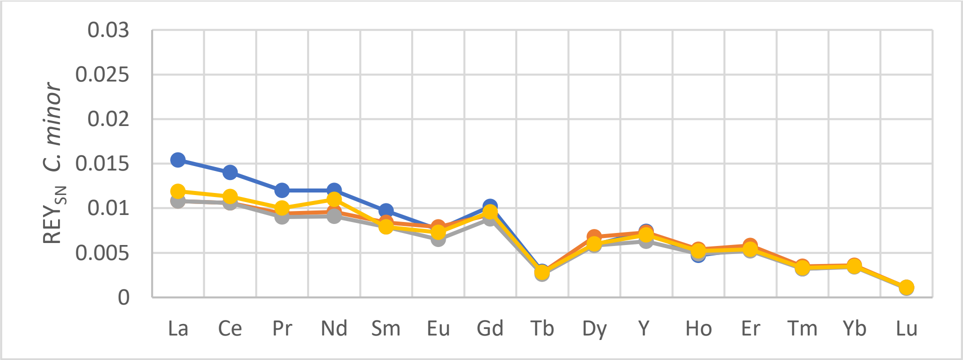

(h)

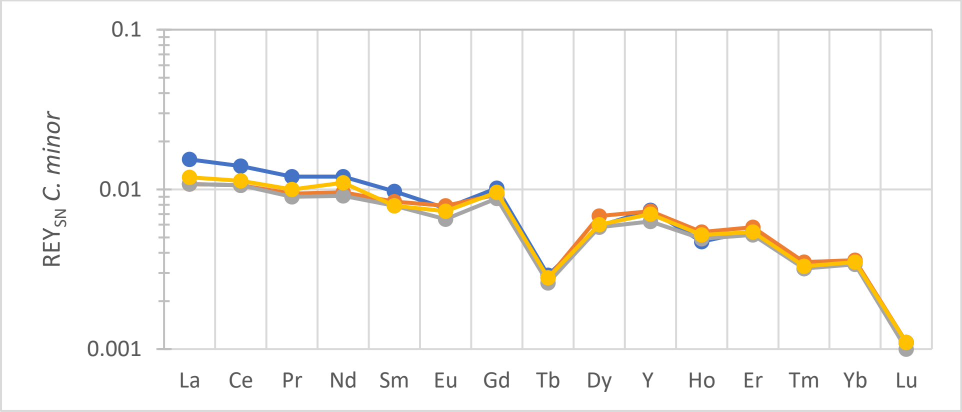

(h´)

Figure 4S. NASC-, PAAS-, EUS- and WSH- shale normal and log-normalized patterns of REY in *Cantharellus cibarius* from all sites jointly in Poland (g & g´; median concentrations) and (h & h´) in *Cantharellus minor* from Yunnan in China.

Table 3S. NASC-, PAAS-, EUS- and WSH-normalized REY concentration levels in *C. cibarius*

REE	REE concentration (mg kg^{-1})				Świetokrzyskie region, Włoszowa (21): Normalization				Tatra Mountains, Zakopane (22): Normalization				Median value: Normalization			
	NASC[a]	PAAS[b]	EUS[c]	WSH[d]	NASC	PAAS	EUS	WSH	NASC	PAAS	EUS	WSH	NASC	PAAS	EUS	WSH
La	31.1	44.54	44.3	40.3	0.0018	0.0013	0.0013	0.0014	0.0045	0.0031	0.0032	0.0035	0.00087	0.00061	0.00061	0.00067
Ce	67.033	88.25	88.5	83.3	0.0014	0.0011	0.0011	0.0011	0.0037	0.0028	0.0028	0.0030	0.00052	0.00040	0.00040	0.00042
Pr	7.9	10.15	10.6	9.54	0.0012	0.00093	0.00089	0.00098	0.0034	0.0027	0.0025	0.0028	0.00029	0.00023	0.00022	0.00024
Nd	30.4	37.32	39.5	31.6	0.00095	0.00078	0.00073	0.00092	0.0032	0.0026	0.0024	0.0030	0.00030	0.00025	0.00023	0.00029
Sm	5.98	6.884	7.30	7.30	0.00077	0.00067	0.00063	0.00063	0.0033	0.0029	0.0027	0.0027	0.00052	0.00045	0.00042	0.00042
Eu	1.253333	1.215	1.48	1.31	0.00088	0.00090	0.00074	0.00084	0.0027	0.0028	0.0023	0.0036	0.00040	0.00041	0.00034	0.00038
Gd	5.5	6.043	6.34	5.86	0.00065	0.00060	0.00057	0.00061	0.0034	0.0031	0.0030	0.0032	0.00071	0.00064	0.00061	0.00066
Tb	0.85	0.891	0.944	0.90	0.00059	0.00056	0.00053	0.00055	0.0098	0.0093	0.0088	0.0092	0.00059	0.00056	0.00052	0.00055
Dy	5.75	5.325	5.86	5.66	0.00042	0.00045	0.00041	0.00042	0.0024	0.0026	0.0024	0.0025	0.00035	0.00038	0.00034	0.00035
Y	27	27.31	31.9	28.7	0.001	0.00099	0.00085	0.00094	0.0031	0.0030	0.0026	0.0029	0.00081	0.00081	0.00069	0.00077
Ho	1.2	1.053	1.17	1.09	0.00042	0.00047	0.00042	0.00046	0.0012	0.0013	0.0012	0.0013	0.00048	0.00055	0.00050	0.00053
Er	3.275	3.075	3.43	3.30	0.00079	0.00084	0.00075	0.00079	0.0024	0.0026	0.0023	0.0024	0.00076	0.00081	0.00073	0.00076
Tm	0.5	0.451	0.492	0.48	0.0010	0.0011	0.0010	0.0010	0.0010	0.0011	0.0010	0.0010	0.0010	0.0011	0.0010	0.0010
Yb	3.113333	3.012	3.26	3.12	0.00016	0.00017	0.00015	0.00016	0.0015	0.0016	0.0015	0.0015	0.00016	0.00017	0.00015	0.00016
Lu	0.456	0.4386	0.485	0.47	0.0011	0.0011	0.0010	0.0011	0.0011	0.0011	0.0010	0.0011	0.0011	0.0011	0.0010	0.0011

Notes: a (North American Shale Composite), b (Post-Archean Australian Shales), c (European Shale), d (World Shale); data for shales from the authors: Bau et al. (2018), Dołęgowska and Migaszewski (2013) and Migaszewski and Gałuszka (2019), respectively.

Table 4S. NASC-, PAAS-, EUS- and WSH-normalized REY concentration levels in *C. cibarius*

REE	REE concentration (mg kg^{-1})				Coastal Landscape Park (ID 1): Normalization				Kaszubski Landscape Park (6): Normalization				Mean: Normalization			
	NASC[a]	PAAS[b]	EUS[c]	WSH[d]	NASC	PAAS	EUS	WSH	NASC	PAAS	EUS	WSH	NASC	PAAS	EUS	WSH
La	31.1	44.54	44.3	40.3	0.0015	0.0010	0.0010	0.0011	0.0019	0.0013	0.0013	0.0015	0.0013	0.00088	0.00088	0.00097
Ce	67.033	88.25	88.5	83.3	0.0014	0.0010	0.0010	0.0011	0.0016	0.0012	0.0012	0.0013	0.00092	0.00070	0.00070	0.00074
Pr	7.9	10.15	10.6	9.54	0.0010	0.00082	0.00078	0.00087	0.0014	0.0011	0.0010	0.0011	0.00068	0.00053	0.00051	0.00057
Nd	30.4	37.32	39.5	31.6	0.00091	0.00091	0.00086	0.0011	0.00095	0.00078	0.00073	0.00092	0.00059	0.00048	0.00046	0.00057
Sm	5.98	6.884	7.30	7.30	0.0015	0.0013	0.0012	0.0012	0.0010	0.00089	0.00084	0.00084	0.00080	0.00070	0.00066	0.00066
Eu	1.253333	1.215	1.48	1.31	0.00096	0.00099	0.00087	0.00092	0.00040	0.00041	0.00034	0.00038	0.00078	0.00080	0.00066	0.00075
Gd	5.5	6.043	6.34	5.86	0.00096	0.00088	0.00084	0.00090	0.00064	0.00058	0.00055	0.00060	0.00040	0.00036	0.00035	0.00037
Tb	0.85	0.891	0.944	0.90	0.00059	0.00056	0.00053	0.00055	0.0012	0.0011	0.0011	0.0011	0.0011	0.0010	0.00095	0.0010
Dy	5.75	5.325	5.86	5.66	0.00052	0.00056	0.00051	0.00053	0.00070	0.00075	0.00068	0.00071	0.00050	0.00054	0.00049	0.00051
Y	27	27.31	31.9	28.7	0.00159	0.00157	0.00135	0.00150	0.00126	0.00124	0.00107	0.00118	0.0012	0.0012	0.0010	0.0011
Ho	1.2	1.053	1.17	1.09	0.00042	0.00047	0.00042	0.00046	0.00042	0.00047	0.00042	0.00046	0.00042	0.00047	0.00042	0.00046
Er	3.275	3.075	3.43	3.30	0.0012	0.0012	0.0011	0.0012	0.0011	0.0012	0.0011	0.0011	0.00095	0.0010	0.00090	0.00094
Tm	0.5	0.451	0.492	0.48	0.0010	0.0011	0.0010	0.0010	0.0010	0.0011	0.0010	0.0010	0.0010	0.0011	0.0010	0.0010
Yb	3.113333	3.012	3.26	3.12	0.00016	0.00017	0.00015	0.00016	0.00016	0.00017	0.00015	0.00016	0.00035	0.00036	0.00034	0.00035
Lu	0.456	0.4386	0.485	0.47	0.0011	0.0011	0.0010	0.0011	0.0011	0.0011	0.0010	0.0011	0.0011	0.0011	0.0010	0.0011

Notes: a (North American Shale Composite), b (Post-Archean Australian Shales), c (European Shale), d (World Shale); data for shales from the authors: Bau et al. (2018), Dołęgowska and Migaszewski (2013) and Migaszewski and Gałuszka (2019), respectively.

Table 5S. NASC-, PAAS-, EUS- and WSH-normalized REY concentration levels in *C. cibarius* and *C. minor*

REE	REE concentration (mg kg^{-1})				Tuchola Pinewoods, Lubichowo (9): Normalization				Augustowska Primeval Forest (15): Normalization				*C. minor*: Normalization			
	NASC[a]	PAAS[b]	EUS[c]	WSH[d]	NASC	PAAS	EUS	WSH	NASC	PAAS	EUS	WSH	NASC	PAAS	EUS	WSH
La	31.1	44.54	44.3	40.3	0.0035	0.0025	0.0025	0.0027	0.0021	0.0015	0.0015	0.0016	0.0154	0.0108	0.0108	0.0119
Ce	67.033	88.25	88.5	83.3	0.0033	0.0030	0.0025	0.0026	0.0017	0.0013	0.0013	0.0014	0.0140	0.0106	0.0106	0.0113
Pr	7.9	10.15	10.6	9.54	0.0029	0.0028	0.0021	0.0023	0.0013	0.00098	0.00099	0.0010	0.0120	0.0094	0.0090	0.0100
Nd	30.4	37.32	39.5	31.6	0.0028	0.0023	0.0021	0.0027	0.0012	0.00097	0.00091	0.0011	0.012	0.0096	0.0091	0.011
Sm	5.98	6.884	7.30	7.30	0.0028	0.0025	0.0023	0.0023	0.0017	0.0014	0.0014	0.0014	0.0097	0.0084	0.0079	0.0079
Eu	1.253333	1.215	1.48	1.31	0.0033	0.0034	0.0028	0.0031	0.0016	0.0016	0.0013	0.0015	0.0076	0.0079	0.0065	0.0073
Gd	5.5	6.043	6.34	5.86	0.0033	0.0030	0.0028	0.0031	0.0010	0.00093	0.00088	0.00096	0.0102	0.0093	0.0088	0.0096
Tb	0.85	0.891	0.944	0.90	0.00059	0.00056	0.00053	0.00055	0.0013	0.0012	0.0012	0.0012	0.0029	0.0028	0.0026	0.0028
Dy	5.75	5.325	5.86	5.66	0.0021	0.0022	0.0020	0.0021	0.0011	0.0011	0.0010	0.0011	0.0059	0.0068	0.0058	0.0060
Y	27	27.31	31.9	28.7	0.0031	0.0030	0.0026	0.0029	0.0021	0.0020	0.0017	0.0019	0.0074	0.0073	0.0063	0.0070
Ho	1.2	1.053	1.17	1.09	0.0011	0.0012	0.0011	0.0012	0.00042	0.00047	0.00042	0.00046	0.0047	0.0054	0.0049	0.0052
Er	3.275	3.075	3.43	3.30	0.0036	0.0027	0.0024	0.0025	0.0018	0.0019	0.0017	0.0018	0.0055	0.0058	0.0052	0.0054
Tm	0.5	0.451	0.492	0.48	0.0010	0.0011	0.0010	0.0010	0.0010	0.0011	0.0010	0.0010	0.0032	0.0035	0.0032	0.0033
Yb	3.113333	3.012	3.26	3.12	0.0018	0.0018	0.0017	0.0018	0.00083	0.00086	0.00080	0.00083	0.0035	0.0036	0.0034	0.0035
Lu	0.456	0.4386	0.485	0.47	0.0011	0.0011	0.0010	0.0011	0.0011	0.0011	0.0010	0.0011	0.0011	0.0011	0.0010	0.0011

Notes: a (North American Shale Composite), b (Post-Archean Australian Shales), c (European Shale), d (World Shale); data for shales from the authors: Bau et al. (2018), Dołęgowska and Migaszewski (2013) and Migaszewski and Gałuszka (2019), respectively.

References

Bau M, Schmidt K, Pack A, Bendel V, Kraemer D (2018) The European shale: an improved data set for normalisation of rare earth element and yttrium concentrations in environmental and biological samples from Europe. Appl Geochem 90:142–149

Dołęgowska S, Migaszewski ZM (2013) Anomalous concentrations of rare earth elements in the moss-soil system from south - central Poland. Environ Poll 178:33-40

Migaszewski ZM, Gałuszka A (2019) Pierwiastki ziem rzadkich w kwaśnych wodach kopalnianych – zarys problematyki. Przegląd Geologiczny, 67, 105-114. doi: http://dx.doi.org/10.7306/2019.2
